# Laparoscopic-assisted cyst excision and ductoplasty plus widened portoenterostomy for choledochal cysts with a narrow portal bile duct

**DOI:** 10.1007/s00464-018-06635-4

**Published:** 2019-01-02

**Authors:** Xiaopan Chang, Xi Zhang, Meng Xiong, Li Yang, Shuai Li, Guoqing Cao, Ying Zhou, Dehua Yang, Shao-tao Tang

**Affiliations:** Department of Pediatric Surgery, Union Hospital, Tongji Medical College, Huazhong University of Science and Technology, Wuhan, 430022 China

**Keywords:** Laparoscopy, Wide hepaticojejunostomy, Anastomotic stricture, Choledochal cyst

## Abstract

**Background:**

Complete cyst excision with Roux-en-Y hepaticojejunostomy is the standard procedure for choledochal cysts (CCs). In recent years, neonates have been increasingly diagnosed with CCs prenatally. Earlier treatment has been recommended to avoid complications. For type IVa malformation without extensive intrahepatic bile duct dilatation, laparoscopic hepaticojejunostomy is technically challenging, and anastomotic stricture is a concern. Therefore, we propose laparoscopic synthetical techniques—laparoscopic excision of cyst and ductoplasty plus widened portoenterostomy to avoid stricture in CCs with a narrow hilar duct.

**Methods:**

An anastomosis was created around the transected end of the common bile duct in 12 minipigs (Group A), and another 12 minipigs (Group B) received conventional cholangiojejunostomy. Anastomotic diameter measurements and cholangiography were conducted at different times. Histological findings of inflammation and scarring were compared. The expression levels of TGF-β1 and type I collagen were detected by real-time quantitative PCR. Between January 2012 and January 2016, laparoscopic excision of cyst and ductoplasty plus widened portoenterostomy were performed on 29 children with confirmed CCs with a narrow portal bile duct who were followed up for 12–48 months.

**Results:**

Group A survived well without obstruction. Slight inflammation and fibrotic tissue were confined to the bile duct periphery. In Group B, five pigs developed stricture. Severe inflammation and diffuse fibrosis affected the whole layer of the anastomosis. Fibrotic biomarkers were significantly higher postoperatively in Group B. Clinically, 29 patients exhibited satisfactory outcomes. No anastomotic stricture has been observed to date.

**Conclusions:**

Laparoscopic synthetical techniques may be a superior option to prevent anastomotic stricture in treating CCs with a narrow portal bile duct.

**Electronic supplementary material:**

The online version of this article (10.1007/s00464-018-06635-4) contains supplementary material, which is available to authorized users.

Complete cyst excision and Roux-en-Y hepaticojejunostomy is the standard procedure for choledochal cysts (CCs) [1–3]. Although the laparoscopic approach has become increasingly popular in the past two decades, advanced skills (especially in hepaticojejunostomy) are required compared with conventional laparotomy [4]. With the development and routine use of prenatal ultrasound screening, more neonates are initially diagnosed with CCs prenatally. Earlier surgical intervention is conducive to preventing complications such as choledocholithiasis, cholangitis and perforation [5]. However, the diameter of the common hepatic duct in neonates is small [6, 7], complicating anastomosis. In addition, type IVa cysts without extensive intrahepatic bile duct dilatation are sometimes encountered in clinical practice. Laparoscopic hepaticojejunostomy is technically challenging in these cases, and the likelihood of anastomotic stricture is high [4–8]. Consequently, the adoption of a modified method to avoid stricture is necessary. In this article, we describe laparoscopic synthetical techniques with a histopathological basis, surgical details and associated clinical effects.

## Methods

### Animal experiments

This work was approved by the Institutional Animal Care and Use Committee of Tongji Medical College, Huazhong University of Science and Technology and conducted in accordance with the guidelines of the Chinese Council on Animal Care in an AAALAC-accredited facility. Twenty-four Bama minipigs (15–20 kg) were randomly divided into Groups A and B (*n* = 12 per group). The animals in each group were randomly numbered from No. 1 to No. 12. Two methods of anastomosis were performed to compare the location and extent of scar formation. Surgery on the animals was carried out by laparotomy due to operative restrictions.

### Operative procedures

After 12 h of fasting, the pigs were anesthetized with an intramuscular injection of 3% pentobarbital sodium (30 mg/kg) and placed in the supine position. Peripheral blood samples were collected from the marginal ear vessels of the 24 pigs, and then the pigs were prepared with 1% povidone iodine and draped with sterile towels. A right upper abdominal transverse incision was made. After the first porta was dissected clearly, we ligated the distal common bile duct with 3-0 silk and transected it. A patch of tissue was obtained from the common bile duct and stored at − 80 °C. An 8–10-cm Roux loop was prepared by transecting the jejunum downstream. Then, a stoma was created on the jejunal limb for later anastomosis.

**Fig. 1:**
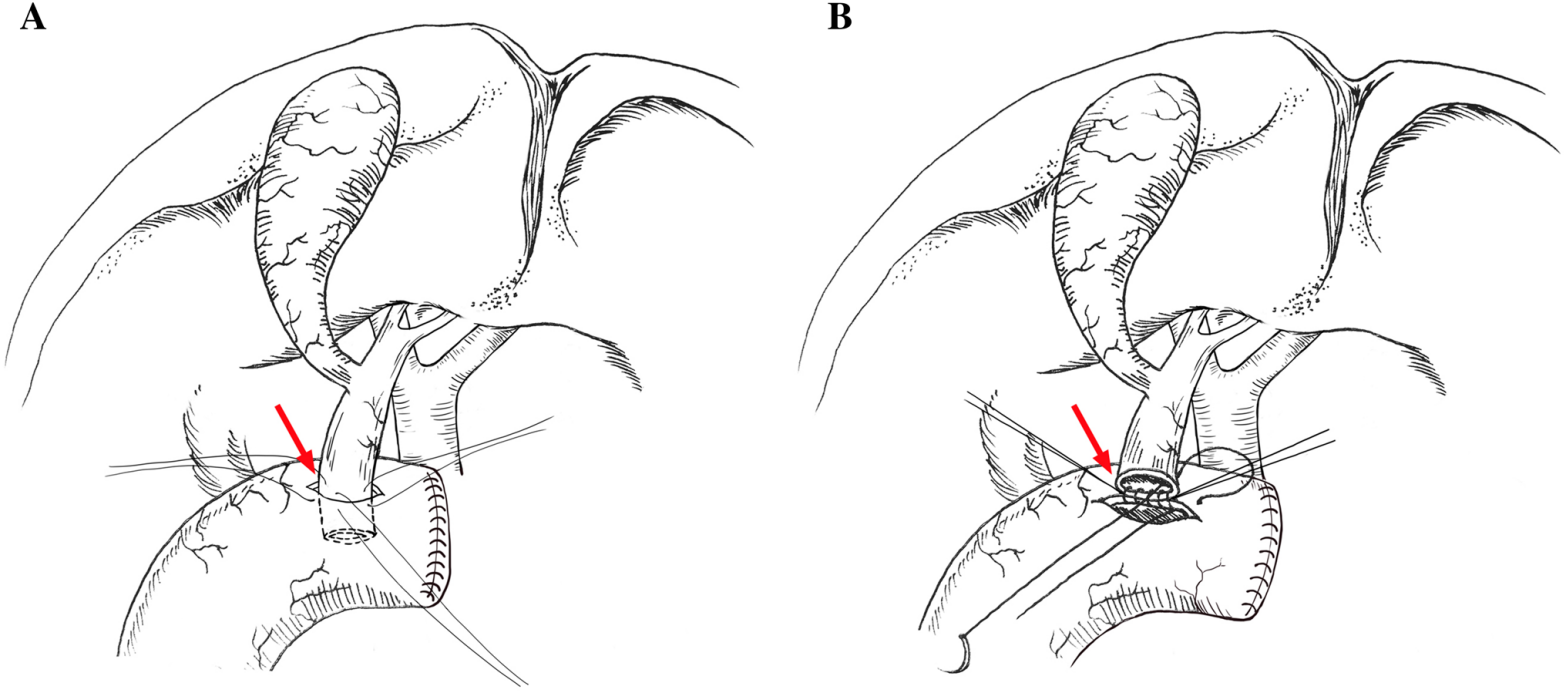
Schematic illustration of two different anastomotic methods in Group A and B: **A** The seromuscular layer of the common bile duct was sutured interruptedly to the whole layer of the jejunum (arrows), simulating the scarring condition of an anastomosis around the transected end of the portal bile duct; **B** The whole layer of the common bile duct was sutured to the whole layer of the jejunum interruptedly (arrows), simulating the scarring condition of the hepaticojejunostomy

In Group A, the bile duct stump was embedded into the jejunal stoma. The seromuscular layer of the common bile duct was sutured interruptedly to the full layer of the jejunum with 5-0 Vicryl (Fig. 1A), simulating the anastomosis around the transected end of the portal bile duct. A conventional end-to-end cholangiojejunostomy was performed in Group B to model the same procedure in clinical practice. We apposed and sutured the full layer of the jejunum and common bile duct interruptedly with 5-0 Vicryl (Fig. 1B). Next, a T tube was inserted into the gallbladder and fixed subcutaneously with 4-0 silk. The abdominal cavity was inspected to ensure the absence of active bleeding before closure. After the operation, ceftazidime (30 mg/kg) was injected intramuscularly for 3 days to prevent infection.

### Biochemical tests

Blood samples were collected from a marginal ear vessel 1 week, 1 month and 3 months after surgery from pig No. 1–4, No. 5–8 and No. 9–12 in each group, respectively. Liver function was tested and compared. A paired *t* test was used for statistical analysis.

### Postoperative cholangiography

Gallbladder cholangiography was conducted 1 week, 1 month and 3 months after surgery when the tissue specimens were collected. Each anastomosis was observed and recorded.

### Tissue harvest and histological test

At each timepoint (1 week, 1 month, and 3 months after surgery), four animals in each group were anesthetized with an intramuscular injection of 3% pentobarbital sodium (30 mg/kg). Specimens of biliary-enteric anastomoses were harvested in vivo, and then the animals were sacrificed by air embolism. After measuring the inner diameter of the anastomotic stoma, a partial sample was stored in RNA Stabilization Reagent (RNA later, Hilden Germany) at − 80 °C. The remaining tissue was fixed in 10% formalin for 24 h and embedded in paraffin. Tissues were sectioned at a thickness of 4 µm and stained by hematoxylin-eosin (H&E, Goodbio Technology, Wuhan China) and Masson’s trichrome (Bogoo Biotechnology, Shanghai China).

### Real-time fluorescence quantitative PCR

Frozen tissue was thawed and treated with TRIzol (Life Technologies, Carlsbad, CA) to obtain total RNA. DNAse digestion and reverse transcription reactions were conducted using the PrimeScript RT reagent kit with gDNA Eraser (Perfect Real Time, Japanese). Then, real-time PCR was performed with SYBR Premix Ex Taq TM (Tli RNaseH Pluse, Japanese). The gene-specific primers for type I collagen and TGF-β1 are listed in the Supplemental Table. The Ct value of each sample was obtained, and the expression levels of the target genes were analyzed by the 2^−ΔΔCt^ method.

## Clinical research

### Patients and methods

Between January 2012 and January 2016, 29 children (22 girls and 7 boys) were diagnosed with a CC with a narrow hilar duct and underwent a laparoscopic synthetical procedure in our center. An informed consent form was signed before surgery. Patient medical records and radiological findings were reviewed retrospectively. The patients’ ages ranged from 3 weeks to 15 years (15 patients were younger than 1 month, two patients were younger than 3 months, seven patients were younger than 6 months, three patients were younger than 1 year, and two patients were older than 1 year). These patients were classified based on ultrasonographical examination and magnetic resonance cholangiopancreatography. Nineteen patients (65.5%) had type Ia cysts and ten patients (34.5%) had type IVa cysts.

### Laparoscopic technique

A conventional four-port method was used to separate the cyst from the distal pancreatic duct to the proximal porta hepatis. A detailed resection of the cyst was performed according to a previously described procedure [9]. For type I cysts with an obvious dilatation of the extrahepatic duct, we split the common hepatic duct upward to achieve a wide anastomosis. In neonates and infants, the diameter of the common hepatic duct is < 3 mm. The opening reaches 4–6 mm at best after the common hepatic duct is split upward until the bilateral bile ducts are exposed. In type IVa, cases with stricture in the porta hepatis, we removed the calculus and flushed the biliary tract after radical excision of the extrahepatic cyst, and the stenotic region was embedded in the shallow hepatic tissue such that the bile duct remnants (4–5 mm) were deeply set.

**Fig. 2:**
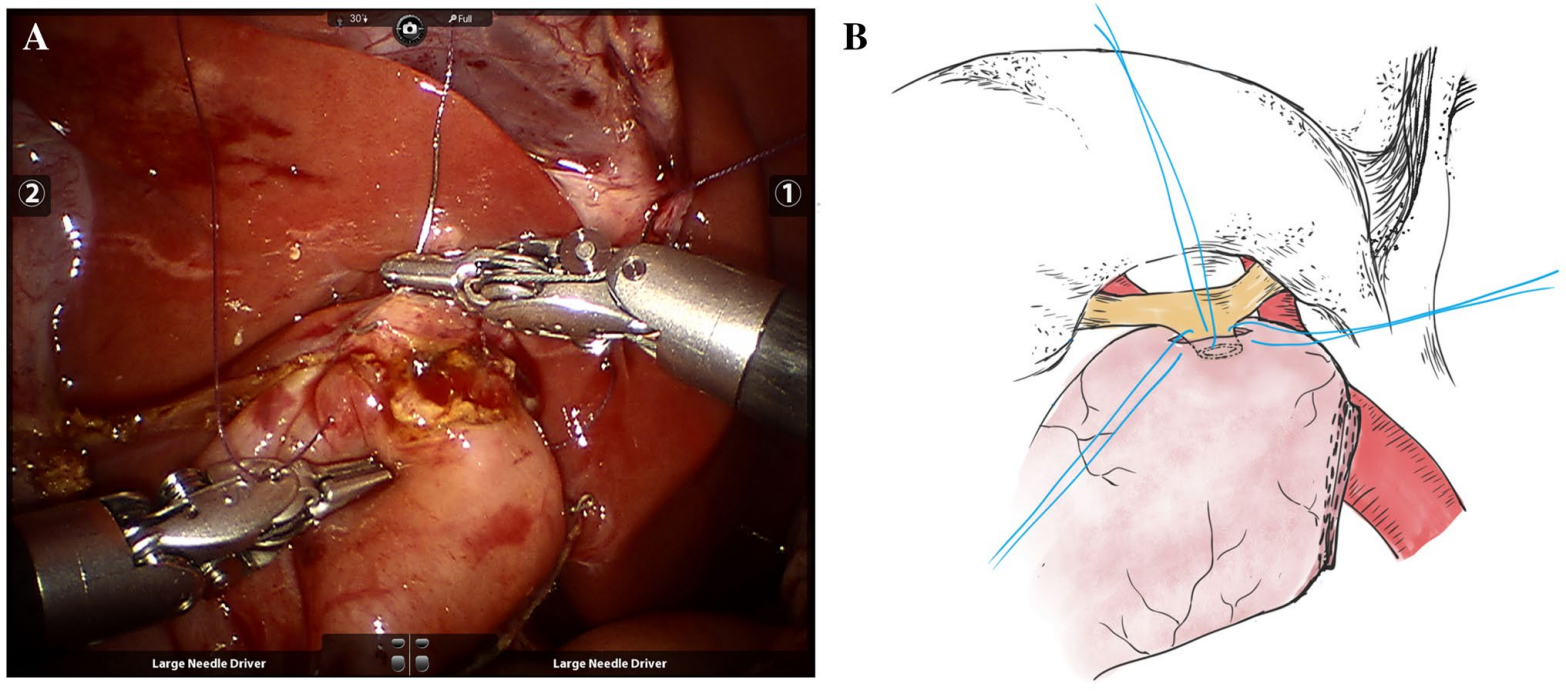
Completed appearance (**A**) and schematic illustration of a laparoscopically widened portoenterostomy (**B**)

Roux-en-Y jejunojejunostomy was performed extracorporeally followed by biliary-enteric anastomosis. The Roux limb was introduced to the porta hepatis from behind the transverse colon, and then a stoma was created in the bowel. The peripheral capsule (Glisson capsule) around the transected end of the portal bile duct was sutured continuously to the full-thickness wall of the intestine, creating a wide opening (10–15 mm). The suture started from the rear wall and traveled to the anterior side, and then the portal bile duct was wrapped in the intestinal lumen (Fig. 2). Suction drainage was placed in the sub-hepatic region before closing the abdominal cavity.

## Results

The animal operations were successfully completed. Before anastomosis, the diameters of the bile duct in Groups A and B were 5.91 ± 0.78 mm and 5.87 ± 1.09 mm, respectively, with no significant difference. Anastomotic strictures occurred in 5/12 of the experimental pigs (3 at 1 month after surgery and 2 at 3 months after surgery) in Group B. They developed jaundice, fatigue, anorexia and emaciation, and strictures were confirmed intraoperatively. Pig No. 9 in Group B died of obstructive suppurative cholangitis within 3 weeks after surgery. No stricture were observed in Group A.

A comparison of liver function is shown in Table 1. The values in Group A remained within the normal range after surgery. In Group B, high total bilirubin (TB) and aspartate aminotransferase (AST) levels and an alkaline phosphatase (ALT) anomaly appeared in 4/11 of the pigs. One week after surgery, the indicators in Group B were normal. One month after surgery, abnormal TB, AST and ALT levels were found in 3/4 of the pigs. Three months after surgery, abnormal TB, AST and ALT levels were found in 1/3 of the pigs (the data for the dead animal were missing).

**Table 1:** Comparison of pre- and post-operative biochemical test value

Biochemical parameter (mean ± SD)	Group	Pre-operation (*n* = 12)	1 Month post-operation (*n* = 6)	3 months post-operation (*n* = 6)
Total bilirubin µmol/l	A	< 0.7	5.4 ± 4.7	3 ± 2.3
	B	< 0.7	127 ± 76^§^	203 ± 85^§,^*
Alanine aminotransferase U/l	A	17 ± 2	17 ± 6	16 ± 5
	B	20 ± 3	85 ± 16 ^§^	151 ± 23^§,^*
Aspartat aminotransferase U/l	A	27 ± 3	20 ± 5	29 ± 4
	B	25 ± 3	67 ± 11 ^§^	207 ± 41^§,^*

*t* test: ^§^*p* < 0.05 versus pre-operation; *p < 0.05 versus 1 month post-operation

**Fig. 3:**
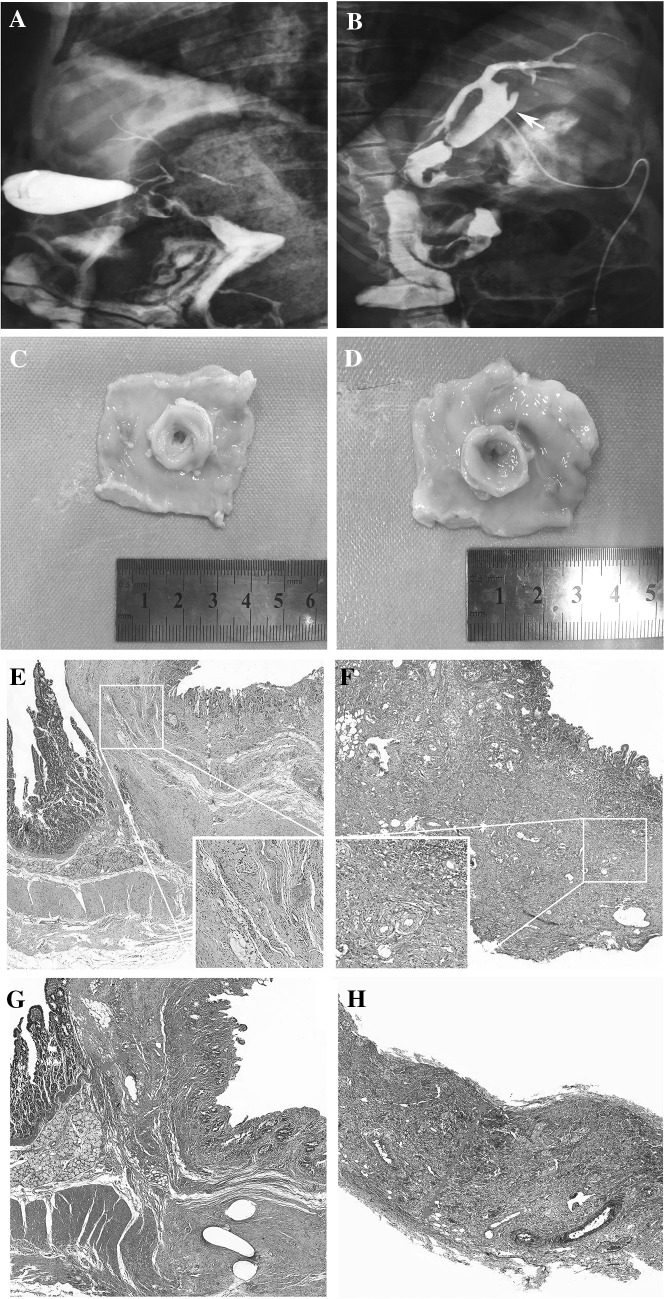
Comparison of the gross and histological findings at 3 months after the operation. **A** Intrahepatic bile ducts and common bile ducts exhibited normal morphology and patency in Group A; **B** Common bile ducts and intrahepatic bile ducts were obviously dilated (arrow) in Group B (4 of 12 pigs); **C** A wider anastomotic stoma (5.58 ± 0.49 mm) in Group A; **D** Anastomotic stenosis (3.00 ± 2.28 mm) in Group B; **E** Anastomotic mucosae healed with slight inflammation in Group A; **F** Severe inflammatory infiltration into the whole layer of the anastomosis was observed in Group B; **G** Thin scar fibers were arranged neatly around the bile duct in Group A (arrow); **H** Coarse mucosae and disordered scar tissue surrounding the whole layer of the anastomosis (arrow) were observed in Group B

Cholangiography at different times revealed no anastomotic stenosis in Group A; the common bile ducts and intrahepatic bile ducts maintained normal morphology and patency (Fig. 3A). In Group B, four living pigs with stricture showed an obviously dilated common bile duct and dilated intrahepatic bile ducts (Fig. 3B), and an anastomotic opening as thin as a needle was found during the operation. The diameter appeared unchanged 1 week after surgery in both groups. One month after surgery, the inner diameters of the anastomotic stoma in Groups A and B were 5.67 ± 0.82 mm and 3.00 ± 2.28 mm (*p* = 0.024), respectively. Three months after the operation, Group A presented a wider opening (5.58 ± 0.49 mm) (Fig. 3C) than did Group B (3.83 ± 1.47 mm) (Fig. 3D) (*p* = 0.038).

According to H&E staining, 1 week after the operation, the mucous membrane was smooth with few inflammatory cells scattered at the periphery of the anastomosis in Group A (Fig. 3E). In sections from Group B, the inner mucous membrane was coarse. Edema and diffuse infiltration of inflammatory cells were evident in the full layer of the anastomosis (Fig. 3F). The first month after surgery was a transition period, during which inflammatory cells in Group B were confined to local tissue. According to Masson trichome staining, 3 months after the operation, thin scar fibers were arranged neatly around the duct in Group A (Fig. 3G). By contrast, disordered scar fibers surrounded the full thickness of the anastomosis in Group B (Fig. 3H).

**Fig. 4:**
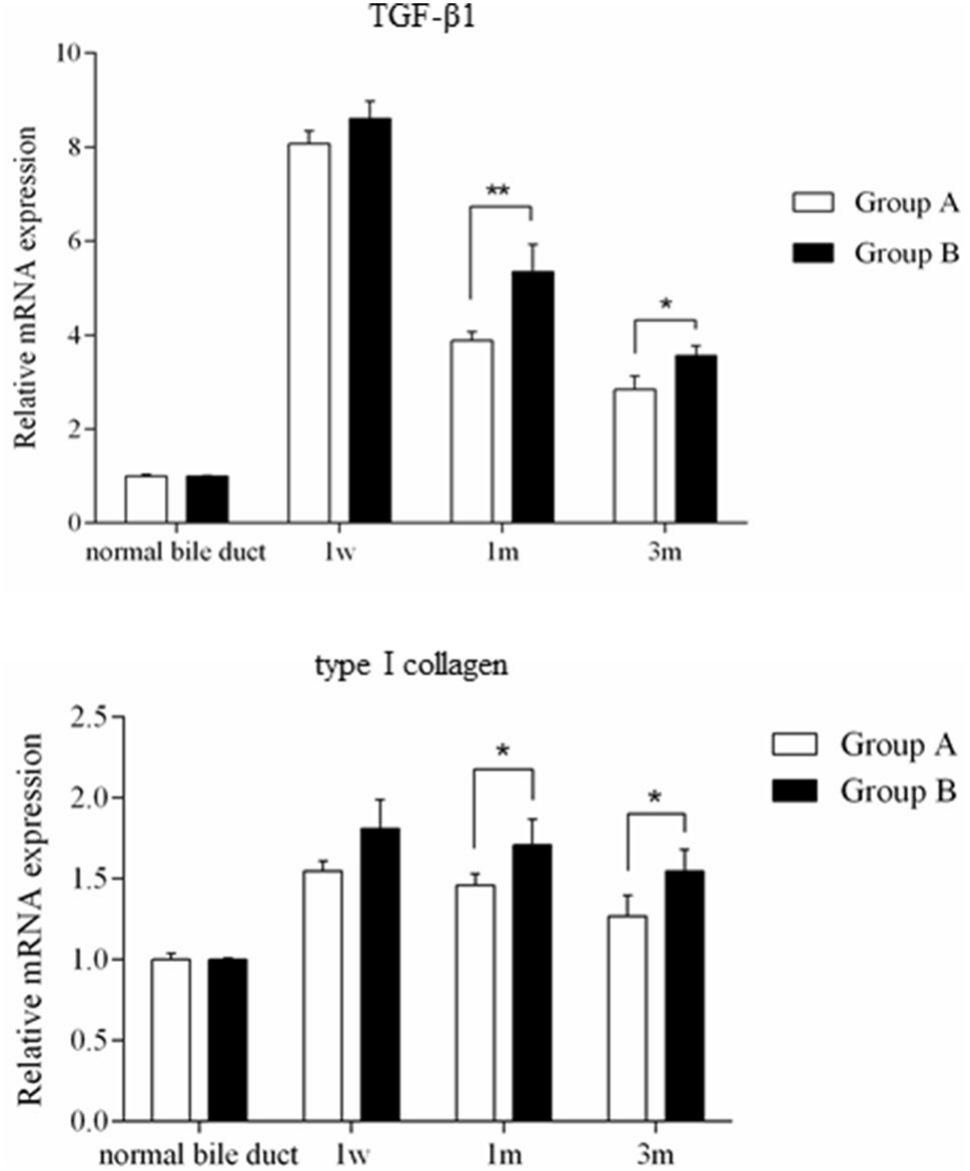
Relative mRNA expression levels of TGF-β1 and type I collagen preoperatively and 1 week, 1 month and 3 months postoperatively

The relative expression level of TGF-β1 peaked at 1 week after surgery and then exhibited a downward trend in both groups (Fig. 4); the peak had no obvious distinction. Significant differences appeared at 1 month and 3 months, revealing that TGF-β1 expression in Group B was higher than that in Group A (*p* < 0.05). Type I collagen mRNA expression also showed obvious differences at 1 and 3 months after surgery; the expression levels in Group B were higher than those in Group A (*p* < 0.05).

Twenty-nine children diagnosed with a CC with a narrow hilar duct were successfully treated surgically using laparoscopic synthetical techniques. The average operative time was 160 min (140–190 min). The amount of bleeding during the operation was approximately 5–10 ml. No anastomotic leakage or other early complications occurred. The average length of the hospital stay was 8.5 days (7–10 days). The average follow-up was 36 months (12–48 months). No intrahepatic stones, cholangitis or anastomotic stricture occurred.

## Discussion

Since Farello et al. [10] first reported laparoscopic-assisted excision of cysts and hepaticojejunostomy in 1995, an increasing number of centers have adopted this technique as a safe, efficacious, and minimally invasive procedure. However, postoperative biliary complications such as bile leakage and anastomotic stricture are major problems [4]. The literature indicates that the rate of anastomotic stricture in open surgery is 6–40% [11–14], while in laparoscopic surgery, it is 0.6–28.6% [2, 3, 15–18]. Our center has used clinical laparoscopic hepaticojejunostomy for the treatment of CCs since 2005 [9]. By January 2016, 396 patients had undergone laparoscopic complete cyst excision, hepaticojejunostomy, and external Roux-en-Y anastomosis in our center. Fifteen children (3.8%) underwent reoperation due to stricture. The average time when stricture appeared is 4.7 years (1–10 years).

Anastomotic stricture reportedly occurs more frequently in cyst types Ia and IVa [13, 16, 19, 20] and is the main reason for reoperation [21, 22]. In 2008, Kim et al. [13] described 34 patients who underwent open hepaticojejunostomy, and anastomotic stricture occurred in eight patients, including seven patients (46.7%) with type IVa cysts. In 2012, Jung et al. [16] described 35 patients with CCs who underwent laparoscopic hepaticojejunostomy; postoperative biliary stricture occurred in ten patients, including seven (53.8%) patients with type Ia cysts. Among the 15 patients who developed strictures in our center, 10 were categorized as type Ia (including 3 neonates), 1 was categorized as type Ic, and 4 were categorized as type IVa. They are also the categories that we concern in this study.

An important association exists between the diameter of the anastomotic opening and the incidence of anastomotic stricture. In 2015, Naoto Urushihara et al. [4] described four patients with a small hepatic duct (diameter < 5 mm) with a stricture requiring correction by easing the anastomosis. Recently, a retrospective study of 275 CC cases in Shanghai, China reported five anastomotic strictures with/without stones between January 1995 and December 2014. The mean diameter of the anastomosis was 4.8 ± 0.8 mm [22]. As previously mentioned, the mean diameter of the common bile duct in the minipigs in this study was 6 mm, which is similar to the duct size in neonates after the left and right hepatic ducts are split. We reviewed the above-mentioned ten children with Ia cyst and postoperative stricture in our center. The diameter of their common hepatic ducts at standard surgery is 4.1 ± 0.9 mm. Although no consensus regarding the optimal luminal diameter for biliary-enteric anastomosis exists, the data above may suggest that an anastomotic diameter of no < 5 mm enables adequate biliary drainage.

Various management options have been proposed for the anastomosis in a CC with a narrow hilar duct, such as hilar ductoplasty with a wide hepaticojejunostomy [23–25], a modified Kasai’s procedure [8], and partial hepatectomy with a wide hepaticojejunostomy [19, 26]. Hilar ductoplasty with hepaticojejunostomy resulted in satisfactory outcomes for type Ia CCs based on a modest sample size after a minimum of 2 years of follow-up, and a reduction of anastomotic strictures was observed compared with conventional hepaticojejunostomy [27]. However, the application of this technique can be difficult under the following circumstances.

For some Ia cysts (especially in neonates), the diameter of the common hepatic duct is only 3 mm or less. Even after splitting the common bile duct upward until the bilateral bile ducts are exposed, the opening only reaches 5–6 mm at best [6, 7], and the anastomotic margin of the ductal tissue is deeply mired in the hilar region. Some doctors reserve part of the dilated cyst wall for convenience and achieve a wide anastomosis (8–10 mm), but carcinoma may develop from the residual cyst wall in the long term [28–31]. Ohashi et al. [32] reported that the risk of malignancy in the remnant bile duct increases after 15 years, postoperatively. Sastry et al. [33] reported that before the age of 18, the incidence of malignancy in CCs is low (0.4%), but in adults, the incidence of malignancy is 11.7%; the peak incidence occurs in the 6th to 7th decades, with a malignancy rate of 38%. Recently, a meta-analysis was conducted by Ten Hove et al. [34] including 2904 patients with a median age of 36 years. The study concluded that malignant transformation was found almost exclusively among those with remnant type I or IV cysts (99%). A malignant transformation rate of 3.4%, mainly in patients who underwent drainage procedures (preservation of the affected biliary tract), is relatively high; therefore, complete surgical resection is recommended. Type IVa malformations with stricture around the hepatic hilum are frequently encountered in clinical practice. After radical excision of an extrahepatic cyst and excavation of stenosis at the hilum, the remaining bile duct stump resides in the deep recesses of the liver tissue. Hepaticojejunostomy under laparoscopy is technically difficult. Therefore, achieving both a complete excision of an extrahepatic lesion and a feasible construction of the anastomosis is necessary in CC cases with a narrow hilar bile duct.

**Fig. 5:**
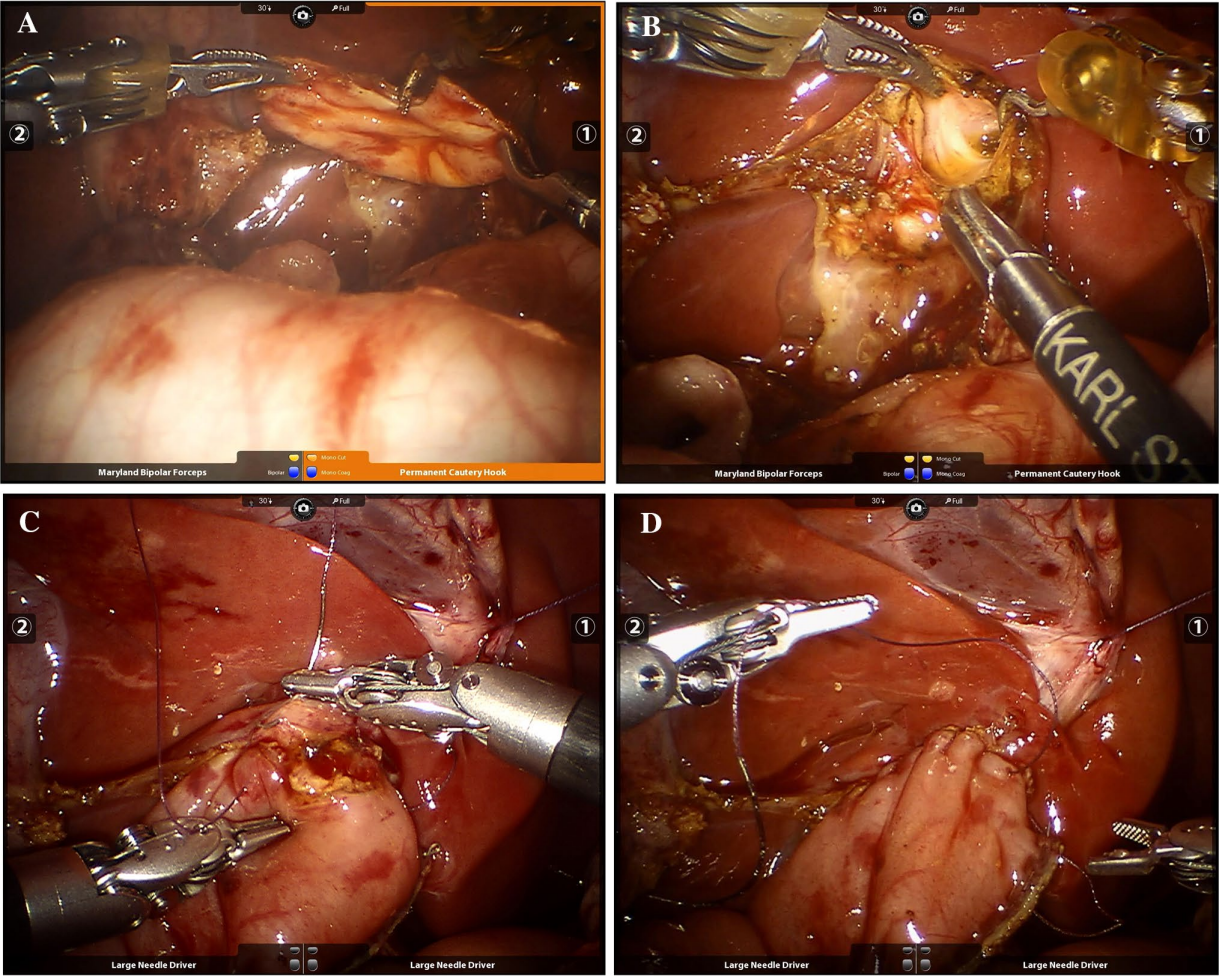
Intraoperative pictures of robotic-assisted laparoscopic synthetical techniques: **A** Complete excision of the cyst; **B** Widening of the opening created by splitting along the bilateral hepatic ducts; **C** Anastomosis around the transected end of the portal bile duct; **D** Completed appearance

Inspired by a report from Morotomi et al. [8], we propose laparoscopic synthetical techniques for the treatment of CCs. Hepatic portojejunostomy with Roux-en-Y anastomosis (Kasai’s procedure) was previously proposed by Morotomi et al. when treating two infants with CCs and narrow hilar ducts (< 3 mm). The laparoscopic synthetical techniques in this study include three key elements—complete excision of the dilated bile duct, widening of the opening by splitting along the bilateral hepatic ducts, and anastomosis around the transected end of the portal bile duct. The technique resembles Kasai’s procedure for the treatment of biliary atresia when the bile duct is unavailable for anastomosis after resection of the fibrotic triangular cord in the porta hepatis. Our center has been practicing robot-assisted surgery since 2015. Furthermore, we used the da Vinci surgical robotic system in the three most recent cases, which enhanced visualization and precision; the same principle as that of the laparoscopic synthetical techniques was followed (Fig. 5). A short intraoperative video segment is included in the supplementary material (video). First, the excision of the dilated bile duct without any remnants clears the inflamed tissue and minimizes the malignancy risk. Then, the enlargement of the hilar bile ducts (hepatic ductoplasty) enables adequate outflow of bile. Finally, the anastomosis around the transected end of the portal bile duct (the connection of the jejunum and the Glisson capsule around the hilar duct) minimizes the associated negative effects caused by the contraction of circular scarring.

Minipigs were selected in this study to confirm the histological basis of this anastomotic option. They exhibit stable heredity features, and their organ function and organizational structures are similar to those in the human body [35]. The finding of five pigs with stricture in Group B reflects worse surgical outcomes with cholangiojejunostomy when the anastomosis is < 6 mm in diameter. The abnormal liver function test results (4/11) in Group B suggest liver damage due to obstruction, and the histological observations indicate a delayed healing phase for anastomotic mucous membranes and a more severe inflammatory reaction involving the full layer of the anastomosis after cholangiojejunostomy. In contrast, an anastomosis around the bile duct stump causes minimal damage to the bile duct, and inflammation is confined to the periphery. After scar remodeling, the anastomotic tissue demonstrates massive and distorted fibers affecting the full layer of the anastomosis after cholangiojejunostomy, while an anastomosis around the bile duct stump results in thin and orderly fibers at the periphery.

Wound healing is characterized by a series of complex matrix–cell interactions involving cellular migration and inflammation, followed by fibroblast proliferation with new collagen synthesis and finally tissue remodeling of the scar. TGF-β1 is a key regulator of the production and remodeling of the extracellular matrix (including type I collagen) [36, 37]. TGF-β1 rapidly reached high levels in both groups at the early proliferative stage. Then, it remained at a high level after cholangiojejunostomy for a fairly long period, leading to more collagen deposition and contraction of the anastomosis, which was confirmed by the overall lower accumulation of type I collagen in the anastomosis around the bile duct stump in the long term.

**Fig. 6:**
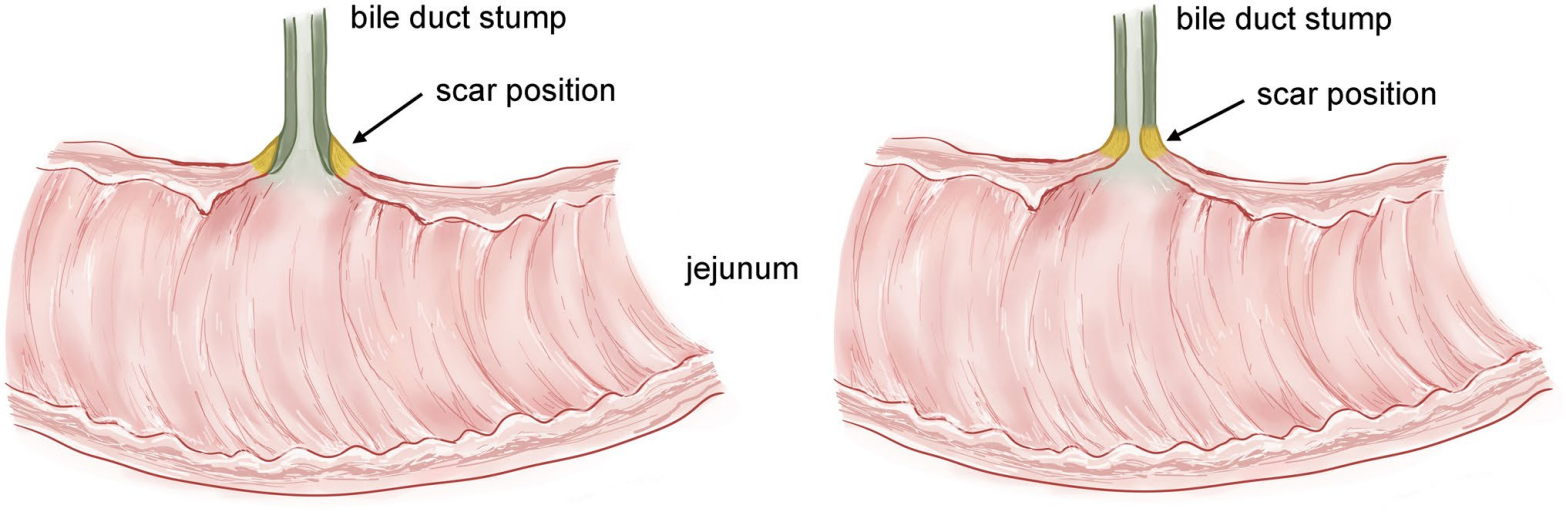
Schematic illustration demonstrating that full layer cicatricial anastomosis after cholangiojejunostomy is more likely to result in stenosis than is anastomosis around the common bile duct stump

Taken together, full layer cicatricial anastomosis after cholangiojejunostomy is more likely to result in stenosis than is anastomosis around the common bile duct stump (Fig. 6). The 5–6-mm-diameter opening contracts to 3–4 mm after remodeling of the circular scarring. However, the 5–6-mm-diameter opening of the bile duct stump is maintained after a widened portoenterostomy. In other words, a widened portoenterostomy is more effective in preventing cicatricial stricture, especially when the opening is < 6 mm in diameter.

Clinically, the application of our synthetical techniques in 29 cases further demonstrates their feasibility and effectiveness in treating CCs with a narrow hilar duct. Moreover, the use of robot-assisted surgery may further enhance the advantages of these techniques. However, a study with a larger sample and a careful long-term follow-up is required to reinforce these results.

## Conclusion

Laparoscopic synthetical techniques may be a superior option to prevent postoperative anastomotic stricture in the treatment of choledochal cysts with an anastomosis < 6 mm in diameter.

## Electronic supplementary material

Below is the link to the electronic supplementary material.

Supplementary material 1 (MP4 35501 KB)

Supplementary material 2 (DOCX 16 KB)

## References

[1] Wen Z, Liang H, Liang J, Liang Q, Xia H (2017). Evaluation of the learning curve of laparoscopic choledochal cyst excision and Roux-en-Y hepaticojejunostomy in children: CUSUM analysis of a single surgeon’s experience. Surg Endosc.

[2] Yeung F, Chung PH, Wong KK, Tam PK (2015). Biliary-enteric reconstruction with hepaticoduodenostomy following laparoscopic excision of choledochal cyst is associated with better postoperative outcomes: a single-centre experience. Pediatr Surg Int.

[3] Senthilnathan P, Patel ND, Nair AS, Nalankilli VP, Vijay A, Palanivelu C (2015). Laparoscopic management of choledochal cyst-technical modifications and outcome analysis. World J Surg.

[4] Urushihara N, Fukumoto K, Nouso H (2015). Hepatic ductoplasty and hepaticojejunostomy to treat narrow common hepatic duct during laparoscopic surgery for choledochal cyst. Pediatr Surg Int.

[5] Diao M, Li L, Cheng W (2012). Timing of surgery for prenatally diagnosed asymptomatic choledochal cysts: a prospective randomized study. J Pediatr Surg.

[6] Hamada Y, Ando H, Kamisawa T (2016). Diagnostic criteria for congenital biliary dilatation 2015. J Hepato-Biliary-Pancreat Sci.

[7] Hernanz-Schulman M, Ambrosino MM, Freeman PC, Quinn CB (1995). Common bile duct in children: sonographic dimensions. Radiology.

[8] Morotomi Y, Todani T, Watanabe Y, Noda T, Otsuka K (1996). Modified Kasai’s procedure for a choledochal cyst with a very narrow hilar duct. Pediatr Surg Int.

[9] Tang ST, Yang Y, Wang Y (2011). Laparoscopic choledochal cyst excision, hepaticojejunostomy, and extracorporeal Roux-en-Y anastomosis: a technical skill and intermediate-term report in 62 cases. Surg Endosc.

[10] Farello GA, Cerofolini A, Rebonato M, Bergamaschi G, Ferrari C, Chiappetta A (1995). Congenital choledochal cyst: video-guided laparoscopic treatment. Surg Laparosc Endosc.

[11] Todani T, Watanabe Y, Urushihara N, Noda T, Morotomi Y (1995). Biliary complications after excisional procedure for choledochal cyst. J Pediatr Surg.

[12] Kim JW, Moon SH, Park DH (2010). Course of choledochal cysts according to the type of treatment. Scand J Gastroenterol.

[13] Kim JH, Choi TY, Han JH (2008). Risk factors of postoperative anastomotic stricture after excision of choledochal cysts with hepaticojejunostomy. J Gastrointest Surg.

[14] Cho MJ, Hwang S, Lee YJ (2011). Surgical experience of 204 cases of adult choledochal cyst disease over 14 years. World J Surg.

[15] Narayanan SK, Chen Y, Narasimhan KL, Cohen RC (2013). Hepaticoduodenostomy versus hepaticojejunostomy after resection of choledochal cyst: a systematic review and meta-analysis. J Pediatr Surg.

[16] Jung K, Han HS, Cho JY, Yoon YS, Hwang DW (2012). Is preoperative subclassification of type I choledochal cyst necessary?. Korean J Radiol.

[17] Qiao G, Li L, Li S (2015). Laparoscopic cyst excision and Roux-Y hepaticojejunostomy for children with choledochal cysts in China: a multicenter study. Surg Endosc.

[18] Wang DC, Liu ZP, Li ZH (2012). Surgical treatment of congenital biliary duct cyst. BMC Gastroenterology.

[19] Dutta HK (2012). Hepatic lobectomy and mucosectomy of intrahepatic cyst for type IV-A choledochal cyst. J Pediatr Surg.

[20] Perdikakis E, Chryssou EG, Koulentaki M, Kouroumalis E, Karantanas A (2011). Assessment of a postoperative anastomotic stricture following correction surgery of a type IVa choledochal cyst using Gd-EOB-DTPA-enhanced magnetic resonance cholangiography. Clin J Gastroenterol.

[21] Diao M, Li L, Cheng W (2016). Recurrence of biliary tract obstructions after primary laparoscopic hepaticojejunostomy in children with choledochal cysts. Surg Endosc.

[22] Sheng Q, Lv Z, Xu W, Xiao X, Liu J, Wu Y (2017). Reoperation after cyst excision with hepaticojejunostomy for choledochal cysts: our experience in 18 cases. Med Sci Monit.

[23] Stringer MD (2007). Wide hilar hepaticojejunostomy: the optimum method of reconstruction after choledochal cyst excision. Pediatr Surg Int.

[24] Urushihara N, Fukuzawa H, Fukumoto K (2011). Totally laparoscopic management of choledochal cyst: Roux-en-Y Jejunojejunostomy and wide hepaticojejunostomy with hilar ductoplasty. J Laparoendosc Adv Surg Tech Part A.

[25] Li S, Wang W, Yu Z, Xu W (2014). Laparoscopically assisted extrahepatic bile duct excision with ductoplasty and a widened hepaticojejunostomy for complicated hepatobiliary dilatation. Pediatr Surg Int.

[26] Pal K, Singh VP, Mitra DK (2009). Partial hepatectomy and total cyst excision is curative for localized type IV-a biliary duct cysts—report of four cases and review of management. Eur J Pediatr Surg.

[27] Xia HT, Liu Y, Yang T, Liang B, Wang J, Dong JH (2017). Better long-term outcomes with hilar ductoplasty and a side-to-side Roux-en-Y hepaticojejunostomy. J Surg Res.

[28] Watanabe Y, Toki A, Todani T (1999). Bile duct cancer developed after cyst excision for choledochal cyst. J Hepato-Biliary-Pancreat Surg.

[29] Liu YB, Wang JW, Devkota KR (2007). Congenital choledochal cysts in adults: twenty-five-year experience. Chin Med J.

[30] Stringer MD (2017). Laparoscopic management of choledochal cysts: is a keyhole view missing the big picture?. Pediatr Surg Int.

[31] Ohtsuka H, Fukase K, Yoshida H (2015). Long-term outcomes after extrahepatic excision of congenital choladocal cysts: 30 years of experience at a single center. Hepato-Gastroenterology.

[32] Ohashi T, Wakai T, Kubota M (2013). Risk of subsequent biliary malignancy in patients undergoing cyst excision for congenital choledochal cysts. J Gastroenterol Hepatol.

[33] Sastry AV, Abbadessa B, Wayne MG, Steele JG, Cooperman AM (2015). What is the incidence of biliary carcinoma in choledochal cysts, when do they develop, and how should it affect management?. World J Surg.

[34] Ten Hove A, de Meijer VE, Hulscher JBF, de Kleine RHJ (2018). Meta-analysis of risk of developing malignancy in congenital choledochal malformation. Br J Surg.

[35] Yin L, Yang H, Li J (2017). Pig models on intestinal development and therapeutics. Amino acids.

[36] Klass BR, Grobbelaar AO, Rolfe KJ (2009). Transforming growth factor beta1 signalling, wound healing and repair: a multifunctional cytokine with clinical implications for wound repair, a delicate balance. Postgrad Med J.

[37] Lichtman MK, Otero-Vinas M, Falanga V (2016). Transforming growth factor beta (TGF-beta) isoforms in wound healing and fibrosis. Wound Repair Regen.

