# Supplementary material for: Laparoscopic-assisted cyst excision and ductoplasty plus widened portoenterostomy for choledochal cysts with a narrow portal bile duct
Source: Surg Endosc. 2019 Jan 2;33(6):1998–2007. doi: 10.1007/s00464-018-06635-4 (PMC6505504; doi:10.1007/s00464-018-06635-4)
Supplement: Supplementary file 2 — Supplementary material 2 (DOCX 16 KB) [file 464_2018_6635_MOESM2_ESM.docx]

**Supplemental Table** The primers used in this study for real-time RT-PCR.

| **Gene name** | | **Forward primer sequence** | | | **Reverse primer sequence** |
| --- | --- | --- | --- | --- | --- |
| **β-actin** | 5’-TGAGAACAGCTGCATCCACTT-3’ | | | 5’ -CGAAGGCAGCTCGGAGTT-3’ | |
| **type I collagen** | 5’-GTGGCCCAGAAGAACTGGTA-3’ | | 5’-CGCCATACTCGAACTGGAAT-3’ | | |
| **TGF-β1** | 5’-CCCCTGTCCATCCCTTTATT-3’ | | 5'-AAGCCCCAGTTCCAATTCTT-3' | | |
